# Trajectories of total and domain-specific physical activity and vascular structure and function in mid-adulthood: the Childhood Determinants of Adult Health study

**DOI:** 10.1093/ehjopen/oeag038

**Published:** 2026-03-18

**Authors:** Jack T Evans, Verity J Cleland, Seana Gall, Terence Dwyer, Alison J Venn, Rachel E Climie

**Affiliations:** Menzies Institute for Medical Research, University of Tasmania, 17 Liverpool St, Hobart, Tasmania 7000, Australia; Menzies Institute for Medical Research, University of Tasmania, 17 Liverpool St, Hobart, Tasmania 7000, Australia; Menzies Institute for Medical Research, University of Tasmania, 17 Liverpool St, Hobart, Tasmania 7000, Australia; School of Clinical Sciences, Monash University, 246 Clayton Rd, Clayton, Victoria 3168, Australia; Nuffield Department of Women’s & Reproductive Health, John Radcliffe Hospital, University of Oxford, Oxford OX3 9DU, UK; Murdoch Children’s Research Institute, Royal Children's Hospital, 50 Flemington Rd, Parkville, Melbourne, Victoria 3052, Australia; Menzies Institute for Medical Research, University of Tasmania, 17 Liverpool St, Hobart, Tasmania 7000, Australia; Menzies Institute for Medical Research, University of Tasmania, 17 Liverpool St, Hobart, Tasmania 7000, Australia; Université Paris Cité, INSERM, U970, Paris Cardiovascular Research Center (PARCC), 56 Rue Leblanc, 75015 Paris, France

**Keywords:** Heart disease risk factors, Physical activity, Cohort studies, Life course perspectives, Paediatrics

## Abstract

**Aims:**

Physical activity is a target for early and ongoing cardiovascular health maintenance. However, relationships between life course trajectories of total activity and all comprising domains (leisure, transport, occupational, domestic) with vascular function and structure have not been examined. This study aimed to determine associations between life course activity trajectories and mid-adulthood vascular structure and function.

**Methods and results:**

Using the Australian Childhood Determinants of Adult Health Study [four time points (ages 9–49 years); 1985 baseline], latent class growth mixture modelling assessed life course trajectories of questionnaire-measured total and domain-specific activity (*n* = 2311). Relationships between trajectories and vascular structure [carotid intima–media thickness (*n* = 914), carotid plaques (*n* = 867)] and vascular function [Young’s elastic modulus (*n* = 765), carotid distensibility (n = 765)] were analysed using log-binomial and multivariable regression adjusted for mid-adulthood body mass index, smoking status, occupation type, area-level socio-economic status, and high- and low-density lipoprotein cholesterol. ‘Consistently high’ leisure activity was associated with reduced risk of plaques (RR = 0.56; 95% CI = 0.23–0.89). ‘High-increasing’ school/occupational activity was associated with higher carotid intima–media thickness (*β* = 0.06; 95% CI = 0.01–0.11). No associations were observed among Young’s elastic modulus, carotid distensibility, or transport and domestic activity.

**Conclusion:**

This study was the first to assess life course trajectories of total and domain-specific activity against vascular structure and function. Findings highlight that maintaining high levels of leisure-time activity across the life course may be associated with better vascular structure in mid-adulthood.

## Introduction

Cardiovascular disease (CVD) remains the leading cause of mortality internationally.^1^ In 2021 alone, 397 000 cardiovascular-related deaths were attributed to inadequate physical activity (PA).^2^ Vascular ageing begins in early life and involves the long-term deterioration of vascular structure and function that may lead to organ damage or overt CVD.^3^ Due to the lifelong nature of vascular ageing and CVD development, PA has been highlighted as a readily accessible target for early and ongoing vascular health maintenance across the life course (from early childhood through late adulthood) due to its dynamic nature.

Despite the clear benefits of PA, globally, 31.3% of adults and 81.0% of children and adolescents do not meet current PA guidelines.^4,5^ PA can be accumulated across four key domains: leisure-time (e.g. sport and exercise), transport (e.g. walking or cycling for transport), domestic (e.g. home maintenance and housework), and occupational PA (e.g. activity undertaken as part of employment). Both child^6^ and adult^7^ physical inactivity has been associated with increased cardiovascular disease risk in adulthood. The benefits of total and leisure-time PA for cardiovascular health and mortality are well-established,^8,9^ and there is growing evidence of an apparent paradox with greater occupational PA associated with higher cardiovascular risk.^9,10^ Comparatively, less is known about the cardiovascular health benefits of transport-related,^11^ or domestic PA,^12^ nor the role of individual PA domains independently. As the global prevalence of physical inactivity remains stagnant and prominent among the world’s youth,^4^ the need to address adverse cardiovascular disease risk at an early stage of disease development has become imperative.

Trajectories of leisure-time and total PA across the life course have observed PA to primarily decline from childhood through adulthood, with small subsamples following increasingly active trajectories.^13,14^ Transport-related PA has been examined to a lesser extent, with trajectories of persistently low and high and increasing PA determined across adulthood, independent of childhood.^15^ In contrast, trajectories of occupational and domestic activity, and their influence on CVD, remain relatively unexplored. Further, there is a sparsity of studies in which all domains of activity are assessed simultaneously alongside vascular measures.^16^ Given that participation in the PA domains may vary across the life course, understanding of PA’s dynamic nature in relation to measures of vascular structure and function (representative of atherosclerosis and arteriosclerosis) may capture associations with the early and often asymptomatic cardiovascular health deterioration and facilitate preventive measures prior to overt CVD development.^17^

Therefore, using the Childhood Determinants of Adult Health (CDAH) study, a population-based cohort of adults followed from childhood in Australia, this study aimed to determine the association between life course trajectories of total and domain-specific PA and vascular structure and function in mid-adulthood.

## Methods

### Study overview

Data were drawn from the Childhood Determinants of Adult Health (CDAH) study, a prospective cohort of Australian adults spanning 34 years. The CDAH study is a continuation of the 1985 Australian Schools Health and Fitness Survey (ASHFS) in which a series of follow-up assessments occurred. ASHFS was a representative sample of 8498 Australian children aged 7–15 years who undertook physical and behavioural assessments. Participants aged 9–15 years (*n* = 6559) completed questionnaires in small groups supervised by trained study data collectors. Procedures describing participant selection at baseline have been outlined in detail elsewhere.^18^ A flowchart detailing CDAH study participation is displayed in Supplementary material online, *Figure S1*.

Ethical approval was granted at baseline by the Directors of Education of each state; schools and parents provided consent while children gave assent. Ethical clearance for CDAH follow-ups was obtained from the Human Research Ethics Committee (formerly the Southern Tasmania Research Ethics Committee). Participants provided written informed consent (CDAH-1: H0008152, CDAH-2: H0010454, CDAH-3: H0013826). A STROBE checklist for cohort studies is presented in Supplementary material online, *Table S1*.

### Total and domain-specific physical activity

#### Childhood physical activity

Self-reported PA data were available for 6559 participants aged 9–15 years who had completed non-validated questionnaires.^19^ Childhood total PA (min/week) was calculated as the sum of childhood leisure-time, transport, and school physical activity. Childhood leisure-time PA (min/week) was determined from the self-reported duration and frequency of discretionary sport or exercise over the past week outside of the school setting. Childhood transport-related PA (min/week) was determined from self-reported frequency and duration of active commutes (walking and cycling) to-and-from school across the previous week. Childhood school PA (min/week) was determined from compulsory self-reported school physical education and school sport.^20^ For each activity, frequency was multiplied by duration to estimate minutes per week, and activities were summed to estimate total weekly physical activity in minutes per week.

#### Adult physical activity

Adult total and domain-specific (leisure, work, transport, and domestic) PA were assessed at each CDAH follow-up using the self-administered long-form International Physical Activity Questionnaire (IPAQ).^21^ For each domain, participants reported activity duration and frequency across the past week, from which minutes per week were derived. Total PA levels were calculated as the sum of activity (min/week) of each domain.

### Adult vascular outcomes

Adult outcomes of vascular structure [carotid intima–media thickness (cIMT) (indicative of subclinical carotid atherosclerotic vascular disease), the presence of carotid plaques (focal wall thickening)] and vascular function [Young’s Elastic Modulus (an estimate of arterial stiffness], and carotid distensibility [a measure of carotid artery elasticity)] were assessed in clinics by trained technicians. To enhance clinical interpretability and align with risk stratification practices, outcomes were dichotomized by risk (low, high) for primary analysis; secondary analysis was performed using continuous outcomes to retain sensitivity. A detailed description of outcome measurement and dichotomous cut-point definitions is presented in the Supplementary material. Briefly, outcomes of high-risk carotid intima–media thickness and Young’s elastic modulus were defined at the ≥90th percentile.^22^ This threshold was selected to identify individuals at the extreme end of the distribution, consistent with previous epidemiological studies examining subclinical vascular risk.^22^ This approach facilitates comparability with prior research and highlights clinically meaningful extremes in the absence of universally accepted cut-points for mid-adulthood.

### Covariates

At baseline, covariates of age, sex (biological), body mass index (from measured height and weight), and teacher-rated scholastic ability were recorded. During adult CDAH follow-ups, age, sex, body mass index, smoking frequency, area-level socio-economic status (from postcode Index of Relative Socio-economic Advantage and Disadvantage), highest education level, occupation type, and high- and low-density lipoprotein levels (HDL-C and LDL-C) were recorded. Detailed methods outlining the assessment of covariates are provided in the Supplementary material. Covariates included in final regression models were selected based on the presence of statistical significance of their association with both the exposure of interest and the outcome, and whether the estimated coefficient of the relationship between exposure and outcomes changed by >10% when the potential confounder was included/excluded.^23^

### Statistical methods

Analyses were performed using R (version 3.5.3; R Foundation for Statistical Computing, Vienna, Austria) and RStudio (Version 1.2.1511; RStudio, Inc., Boston, MA, USA) or STATA Version 18.0 (StataCorp LP, College Station, TX, USA).

#### Latent physical activity trajectory identification

Latent class growth mixture modelling (LCGMM), a form of trajectory analysis, was performed to determine trajectories for total PA and each of the four comprising domains (leisure, occupational, transport, and domestic) across the life course using the lcmm 1.9.3 package in R. LCGMM allows for the data-driven identification of trajectories, considering that a population consists of heterogeneous subgroups, each with its own distinct patterns.^24^ Data is used to infer the number of trajectories, their shape across the life course, and the number of participants belonging to each trajectory. Through the integration of residuals into the LCGMM technique, adjustment for time-varying covariates was performed,^15^ a crucial requirement for the analysis of PA trajectories where covariates influence the repeated measures of PA at each time point. Covariates of age, sex, BMI, education, and occupation at each time point across the life course were first identified from prior literature and confirmed as confounders (using the approach detailed above) prior to trajectory analysis.

Trajectory analysis of total PA and the domains of PA (leisure, school/occupational, and transport) was performed across the life course. Childhood school and adult occupational PA were used to create school/occupational trajectories due to their shared compulsory nature—with both undertaken as a requirement of a role (student/employee) and within a structured setting. Domestic PA was only assessed across adult time points, due to a lack of a corresponding childhood variable for this domain. LCGMM was performed among participants with observations at childhood baseline (ASHFS) and a minimum of one of the three adult time points (CDAH-1, CDAH-2, and CDAH-3). Model building and class enumeration were performed in line with the recommendations of Proust-Lima *et al*.^25^ Further detail regarding LCGMM, selection of model specifications, and number of classes is detailed in the Supplementary material. Distinct trajectories of PA (adjusting for covariates of age, sex, education, and occupation) were identified among total and domain-specific life course PA. The latent PA trajectories were qualitatively labelled. PA levels within each PA trajectory are presented in Supplementary material online, *Table S2*.

#### Association of total and domain-specific physical activity trajectories with adult vascular outcomes

For the primary analysis, the association between total and domain-specific PA trajectories and dichotomous vascular structure and function outcomes was assessed via log-binomial regression. Log-binomial regression (binomial errors and log-link) using the ‘breg’ package in STATA provides a practical approach to the determination of multivariable-adjusted estimates of risk ratios, while overcoming issues of substantial overestimation commonly associated with logistic regression using a canonical logit link.^26^ For the secondary analysis, multivariable linear regression was performed using each trajectory of total and domain-specific PA as exposures and continuous outcomes of carotid intima–media thickness (mm), Young’s elastic modulus (mmHg.mm), and carotid distensibility (%/10 mmHg). A continuous measure of carotid plaques was not assessed due to the low prevalence of more than one plaque among participants [*n* = 21 (2.4%)]. LCGMM determined trajectories were introduced as predictors of each adult cardiovascular outcome. Model 1 was adjusted for covariates of age and sex in mid-adulthood, as well as other domains of physical activity. Model 2 was additionally adjusted for mid-adulthood body mass index, smoking status, occupation type, area-level socio-economic status, HDL-C, and LDL-C. A *P*-value of <0.05 was considered statistically significant. Analysis considering only the trajectories of participants with observations at all time points across the life course was also undertaken and presented in the Supplementary material; however, as these analyses were restricted in sample size, the results cannot be interpreted with confidence. Similarly, sensitivity analysis was performed among those with complete-case outcome measures.

## Results

### Participant characteristics

**Figure 1:**
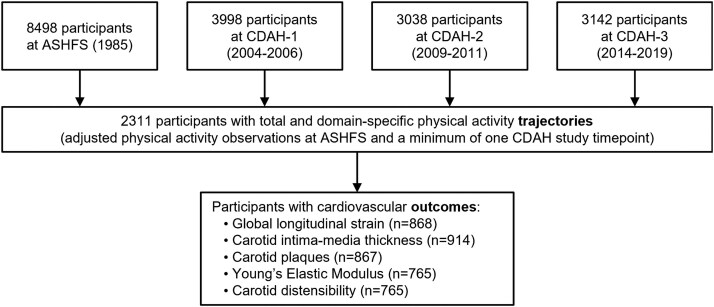
Flow chart of Australian Childhood Determinants of Adult Health Study participants included within this analysis.

Trajectory analysis was performed among 2311 participants with PA observations at baseline (ASHFS) and at least one other adult CDAH time point (*Figure 1*). Participant characteristics are presented within *Table 1*. The mean participant age at baseline (ASHFS) was 12.0 (2.0) years. During adulthood, the mean age of participants was 32.3 (2.1) years at CDAH-1, 37.5 (2.0) years at CDAH-2, and 45.0 (2.2) years at CDAH-3. Participants were more commonly female (52.5–57.8%), were non-smokers (55.8–89.7%), had high education levels (41.8–50.3%), and were employed in professional/managerial occupations (54.1–60.4%).

**Table 1:** Characteristics of participants from which physical activity trajectories were derived

	ASHFS *N* = 2311	CDAH-1 *N* = 1761	CDAH-2 *N* = 1888	CDAH-3 *N* = 1518
Age (years), mean (SD)	12.0 (2.0)	32.3 (2.1)	37.5 (2.0)	45.0 (2.2)
Sex (male), % (*n*)	45.0 (1041)	47.5 (837)	42.2 (796)	45.5 (691)
Body mass index (kg/m^2^), mean (SD)	18.5 (2.7)	25.8 (4.8)	26.2 (5.0)	27.6 (5.7)
HDL (mmol/L), mean (SD)				1.50 (0.40)
LDL (mmol/L), mean (SD)				3.08 (0.81)
Teacher-rated scholastic ability, % (*n*)				
Excellent	13.3 (289)			
Very good	34.9 (756)			
Good	38.9 (843)			
Fair or poor	12.9 (280)			
Highest education level, % (*n*)				
High		41.8 (918)	45.4 (854)	50.3 (764)
Medium		30.6 (672)	31.3 (589)	46.2 (702)
Low		27.5 (604)	23.3 (439)	3.5 (53)
Occupation, % (*n*)				
Managers/professionals		54.1 (1073)	55.3 (748)	61.8 (935)
White collar		16.9 (335)	18.0 (243)	16.5 (250)
Blue collar		16.3 (324)	13.0 (176)	12.9 (196)
Not in labour force		12.7 (251)	13.7 (185)	8.8 (133)
Smoking frequency, % (*n*)				
Never		55.8 (1221)	59.3 (803)	60.4 (891)
Ex-smoker		23.1 (507)	25.9 (351)	29.5 (435)
Less than weekly		3.5 (77)	2.4 (32)	2.0 (29)
Weekly		2.5 (54)	1.3 (18)	1.4 (21)
Daily		15.1 (331)	11.1 (151)	6.7 (98)
Area-level socio-economic status (IRSAD), mean (SD)		1031.9 (88.6)	1019.6 (108.7)	1033.6 (86.7)
Physical activity, median (IQR)				
Total (min/week)	325.0 (190.0, 555.0)	660.0 (370.0, 1070.4)	690.0 (385.0, 1080.0)	705.2 (410, 1117.6)
Leisure-time (min/week)	120.0 (24.0, 300.0)	100.0 (0.0, 240.0)	120.0 (20.0, 240.0)	131.4 (30.0, 270.0)
Transport-related (min/week)	20.0 (0.0, 75.0)	50.0 (0.0, 140.0)	45.0 (0.0, 150.)	60.0 (0.0, 175.0)
Occupational (min/week)	120.0 (60.0, 180.0)	20.0 (0.0, 395.1)	0.0 (0.0, 300.0)	15.0 (0.0, 342.2)
Domestic (min/week)		161.5 (60.0, 360.0)	201.2 (90.0, 450.0)	180.0 (75.0, 360.0)
Young’s elastic modulus (mmHg.mm), mean (SD)				352.1 (142.9)
High-risk (≥90th percentile), % (*n*)				10.2 (79)
Carotid distensibility (%/10 mmHg), mean (SD)				2.02 (0.69)
High-risk (≥90th percentile), % (*n*)				9.75 (75)
Carotid intima media thickness (mm), mean (SD)				0.650 (0.109)
High-risk (≥90th percentile), % (*n*)				11.2 (104)
Carotid plaques present, % (*n*)				13.4 (117)

ASHFS, Australian Schools Health and Fitness Survey; BP, blood pressure; CDAH, Childhood Determinants of Adult Health Study; HDL, high-density lipoprotein; IQR, interquartile range; IRSAD, Index or Relative Socio-economic Advantage and Disadvantage; LDL, low-density lipoprotein; PA, physical activity; SD, standard deviation. N for variables within each time point varies.

### Latent physical activity trajectories

#### Total physical activity

From childhood through adulthood, three distinct trajectories of total PA were observed. A ‘persistently low’ life course trajectory was observed among 21.6% (*n* = 498) of participants, a ‘low to high’ trajectory in 73.5% (*n* = 1669, a trajectory that increased from low levels of activity in childhood to high levels of activity in mid-adulthood), and a ‘consistently high’ trajectory in 4.9% (*n* = 114).

#### Leisure-time physical activity

Three trajectories of leisure-time PA were observed across the life course. Additionally, 85.5% of participants (*n* = 1975) undertook ‘persistently low’ levels of leisure-time activity from childhood to mid-adulthood. A trajectory of very high childhood leisure-time activity that decreased to high levels of adult activity (‘high and decreasing) was observed among 2.2% (*n* = 51), while a third trajectory of ‘consistently high’ leisure-time activity across the life course was observed among 12.3% (*n* = 285) of participants.

#### Transport-related physical activity

Two trajectories of transport-related PA were observed across the life course; 87.7% (*n* = 2026) of participants had ‘persistently low’ trajectories of transport-related activity while 12.3% (*n* = 285) had ‘high and increasing’ patterns of transport-related PA.

#### School/occupational physical activity

Three distinct trajectories of childhood school-based and adulthood occupational PA were identified; 83.4% (*n* = 1928) of participants followed a ‘persistently low’ trajectory of school-based/occupational PA, 13.1% (*n* = 303) followed trajectories from low school-based activity through to high levels of occupation activity (a ‘low to high’ trajectory) in adulthood, and 3.5% (*n* = 80) followed a trajectory of ‘high and increasing’ activity, where high levels were observed during childhood and continued to increase across the life course to the workplace in mid-adulthood.

#### Domestic physical activity

Trajectories of domestic PA were only available across the adult life-period. Two trajectories were observed: 78.8% (*n* = 1821) of participants performed ‘persistently low’ levels of domestic activity across adulthood; contrastingly, 21.2% (*n* = 490) undertook ‘consistently high’ levels of domestic PA from early through mid-adulthood.

### Association of physical activity trajectories with adult vascular outcomes

The analysis of associations between PA trajectories and dichotomous outcomes of vascular structure [high-risk cIMT (*n* = 914), presence of carotid plaques (*n* = 867)] and vascular function [high-risk YEM (*n* = 765), high-risk carotid distensibility (*n* = 765)] is presented in *Tables 2* and *3* and Supplementary material online, *Tables S3*–*S5*. As most participants were classified into the lowest PA trajectory for each domain, the ‘persistently low’ PA trajectories were assigned as the reference group against which the alternate PA trajectories were compared.

**Table 2:** Associations between total physical activity trajectories and dichotomous adult vascular structure and function outcomes

Outcome, latent total physical activity trajectory	Model 1		Model 2	
	*n*/*N*	RR (95% CI)	n/N	RR (95% CI)
High-risk Young’s elastic modulus	79/765		76/713	
Persistently low		REF		REF
Low to high		1.396 (0.758, 2.033)		1.331 (0.683, 1.977)
Consistently high		1.611 (0.183, 3.039)		1.460 (0.157, 2.763)
High-risk carotid distensibility	75/765		72/713	
Persistently low		REF		REF
Low to high		0.940 (0.433, 1.448)		0.915 (0.403, 1.428)
Consistently high		1.985 (0.469, 3.501)		1.878 (0.414, 3.342)
High-risk cIMT	104/914		97/853	
Persistently low		REF		REF
Low to high		1.120 (0.655, 1.591)		1.119 (0.630, 1.608)
Consistently high		1.117 (0.098, 2.136)		1.043 (0.060, 2.026)
Presence of carotid plaques	116/867		110/807	
Persistently low		REF		REF
Low to high		1.071 (0.634, 1.507)		1.146 (0.671, 1.620)
Consistently high		1.653 (0.533, 2.773)		1.453 (0.483, 2.423)

*n*, number with high-risk outcomes; *N*, number with outcomes assessed; RR, relative risk; CI, confidence interval; cIMT, carotid intima–media thickness.

Model 1: adjusted for age and sex.

Model 2: additionally adjusted for occupation-type, body mass index, smoking status, high-density lipoprotein, low-density lipoprotein, and area-level socio-economic status.

**Table 3:** Associations between leisure-time physical activity trajectories and dichotomous adult vascular structure and function outcomes

Outcome, latent leisure-time physical activity trajectory	Model 1		Model 2	
	*n*/*N*	RR (95% CI)	n/N	RR (95% CI)
High-risk Young’s elastic modulus	79/765		76/713	
Persistently low		REF		REF
High and decreasing		NA		NA
Consistently high		1.133 (0.549, 1.718)		1.210 (0.536, 1.886)
High-risk carotid distensibility	75/765		72/713	
High and decreasing		REF		REF
High to low		0.772 (−0.654, 2.198)		1.408 (−1.309, 4.126)
Consistently high		1.026 (0.452, 1.601)		1.053 (0.434, 1.671)
High-risk cIMT	104/914		97/853	
Persistently low		REF		REF
High and decreasing		1.757 (0.067, 3.448)		2.305 (0.179, 4.432)
Consistently high		0.969 (0.464, 1.472)		0.842 (0.360, 1.324)
Presence of carotid plaques	116/867		110/807	
Persistently low		REF		REF
High and decreasing		1.020 (−0.282, 2.312)		1.831 (−0.459, 4.211)
Consistently high		**0.560 (0.228, 0.891)**		**0.587 (0.239, 0.935)**

Bold indicates significance.

*n*, number with high-risk outcomes; *N*, number with outcomes assessed; RR, relative risk; CI, confidence interval; cIMT, carotid intima–media thickness.

Model 1: adjusted for age, sex, and transport, occupational, and domestic physical activity.

Model 2: additionally adjusted for occupation-type, body mass index, smoking status, high-density lipoprotein, low-density lipoprotein, and area-level socio-economic status.

#### Vascular structure

##### Carotid intima–media thickness

Total and domain-specific PA trajectories were not associated with categorical high-risk cIMT (*Tables 2* and *3* and Supplementary material online, *Tables S3*–*S5*). Continuous measures of cIMT were 0.02 mm higher (*β* = 0.020; 95% CI = 0.003, 0.036) among those in the ‘low to high’ trajectory compared to those with ‘persistently low’ total PA trajectory (see Supplementary material online, *Table S6*, Model 2), although this relationship was not retained in the analysis of a subset of participants with complete-case outcome measures (see Supplementary material online, *Table S7*). Those with trajectories of ‘high to very low’ levels of occupational activity in mid-adulthood had 0.06 mm higher cIMT than those with persistently low occupational activity across the life course (*β* = 0.057; 95% CI = 0.004, 0.111) (see Supplementary material online, *Table S8*, Model 2); this relationship was not retained in the analysis of a subset of participants with complete-case outcome measures (see Supplementary material online, *Table S9*).

##### Presence of carotid plaques

‘Consistently high’ leisure-time PA was associated with a 44.0% reduction in the risk of carotid plaques in mid-adulthood compared to ‘persistently low’ leisure-time physical activity in Model 1 (RR = 0.560; 95% CI = 0.228, 0.891). Slight attenuation of effect was observed following further adjustment in Model 2; however, a statistically significant 41.3% reduction of risk remained (RR = 0.587; 95% CI = 0.239, 0.935, *Table 3*). This relationship remained when assessed among participants with complete-case outcome assessments (see Supplementary material online, *Table S10*).

#### Vascular function

##### Young’s elastic modulus

Trajectories of total and domain-specific PA were not associated with categorical or continuous measures of Young’s elastic modulus (*P* > 0.05 for all, *Tables 2* and *3* and Supplementary material online, *Tables S3*–*S5*).

##### Carotid distensibility

No significant relationship was observed between trajectories of total or domain-specific PA domains and categorical or continuous outcomes of carotid distensibility (*Tables 2* and *3* and Supplementary material online, *Tables S3*–*S5*).

## Discussion

Following a sample of Australian children through to adulthood, this study is the first to define life course trajectories of total and domain-specific PA (leisure, transport, school/occupational, domestic) among a single cohort. We also examined the relationship between these specific trajectories and outcomes of vascular structure and function in mid-adulthood. The main finding was that trajectories of total, leisure-time, and school/occupational PA, but not transport-related or domestic PA, were associated with some cardiovascular structure (cIMT, plaques) outcomes. This study suggests the importance of maintaining high levels of total and leisure-time physical activity consistently throughout the life course for healthy vasculature and reduced atherosclerotic risk in adulthood.

### Vascular structure

#### Total physical activity

Our study observed three distinct trajectories of total PA across the life course (persistently low, low to high, and consistently high) and positively associated with cIMT in mid-adulthood. We found that participants with trajectories of low childhood PA increasing to high mid-adulthood PA had less favourable cIMT than participants with ‘persistently low’ trajectories of total PA, which was an unexpected finding. cIMT is associated with CVD events such as stroke,^27^ myocardial infarction,^28^ and the presence and degree of atherosclerosis.^29^ Prior relationships between higher PA levels and lower CIMT^30,31^ have been observed. The explanation for this unexpected finding is unclear, although we hypothesize that greater levels of occupational activity among the ‘low to high’ total PA trajectory may have been responsible. Following sensitivity analysis in which occupational activity was adjusted for, no change in the relationship was observed (data not shown). However, sensitivity analysis among those with measures of all outcomes saw no significant relationship between ‘low to high’ total activity and cIMT, suggesting a small number of outliers may be responsible. These findings indicate the potential for effect via external factors or outlying observations and highlight a need for replication in other datasets to disentangle this observation.

#### Leisure-time physical activity

Three distinct trajectories of leisure-time PA were determined across the life course: ‘persistently low’, ‘high and decreasing’, and ‘consistently high’. Our findings indicate that ‘consistently high’ leisure-time PA across the life course was associated with significantly lower risk of carotid plaques in mid-adulthood compared to persistently low leisure PA. However, associations were not identified among any of the other vascular outcomes assessed. While early-life leisure PA has been associated with decreased vascular disease risk,^32^ we found no significant difference in cardiovascular health outcomes between the ‘persistently low’ trajectories and ‘high and decreasing’ leisure PA. This indicates that maintaining leisure PA beyond childhood may be crucial for vascular benefits in mid-adulthood. Further, it has previously been hypothesized that high PA may decrease associated cardiovascular risk factors (e.g. overweight and obesity), as well as improve vascular repair.^33^ When considering the pathogenesis of plaque development, endothelial injury via oxidative stress is believed to play a key role.^34^ High leisure-time PA has been shown to yield anti-inflammatory properties and protect against oxidative stress, thus slowing vascular damage. Given that vascular aging is a lifelong process, it is plausible that maintained leisure-time activity across the life course may have the greatest effect in slowing the progression of early vascular aging.

#### School/occupational physical activity

We observed three distinct trajectories of school/occupational activity across the life course: ‘persistently low’, ‘low to high’, and ‘high and increasing’. Our analysis suggests that prolonged exposure to higher levels of school/occupational PA across the life course may be associated with higher cIMT and therefore greater cardiovascular disease risk. These findings are supported by prior observations of positive longitudinal relationships between adult occupational PA and IMT.^35^ Further, it is well-established that a paradox exists in which greater occupational PA is associated with higher cardiovascular disease risk.^9,10^ Notably, a significant association was only observed with continuous measures of cIMT, suggesting that classification in a more favourable trajectory of school/occupational activity was not associated with a level of effect sufficient to raise cIMT levels into the highest 10% of the sample population (high-risk cIMT). Our observations are unsurprising as occupational PA is characterized by low intensity, long duration, and low discretion activity. This activity is commonly experienced among workers who experience long working hours and strenuous manual labour that has been associated with systemic inflammation.^9,36^ Further, those undertaking blue-collar work are more likely to be male, receive lower incomes, have less access to resources, live in low socio-economic status areas, and have poorer access to health services.^37^ These factors have the potential to confound or negate the usual benefits of PA. Despite adjusting for BMI, smoking, cholesterol, occupation, and area-level socio-economic status, disentangling the effect of occupational activity from other accompanying lifestyle and socio-economic factors present among those undertaking high levels has been acknowledged as difficult.^38^ There is strong evidence to support the role of school-based physical activity interventions in the short-term reduction of cardiovascular disease risk factors,^39^ but the longitudinal retention of cardiovascular benefits from such activity is not yet certain.^20^ As such, it is possible that the benefits of high school-based activity levels may be offset by the poor cardiovascular associations of adult occupational activity. However, as there is little research into the domain-specific mechanisms between PA and the cardiovascular dysfunction, further research is recommended to shed light on these findings.

#### Transport-related physical activity

Two distinct trajectories of transport-related PA were observed across the life course from childhood to adulthood. These findings align with our previous research on the same cohort of Australian children followed into mid-adulthood, where we identified two distinct classes of adult transport-related PA.^15^ Although represented as a continued pattern of activity from childhood through to adulthood, it must be noted that in line with our prior findings, childhood transport-related PA did not differentiate trajectories of transport-related PA in adulthood. This is observed via a negligible difference in median transport-related PA (and overlap of interquartile range) in childhood when stratified by trajectory (see Supplementary material online, *Table S2*).

We found no significant differences between each trajectory and its relationship with cardiovascular measures. This lack of relationship may be related to limitations of using the IPAQ-L self-report tool to record transport-related PA levels. As transport-related PA typically occurs in short bursts and bouts of activity <10 min in duration are not recorded by the IPAQ, it is possible that participants of the ‘persistently low’ trajectory cumulatively undertook levels of transport-related PA comparable to the ‘high and increasing’ group via unreported smaller (<10 min) and more frequent bouts. Furthermore, studies have shown transport-related PA to be influenced by environmental factors such as distance to destination and land-use mix.^40^ Similarly, at baseline, only activity undertaken when walking or cycling to school was assessed, which may not be completely representative of all transport-related PA undertaken. To further determine the benefits of transport-related PA beyond its capacity to bolster total levels of PA, future research is recommended to determine the domain-specific effects of active travel on CVD.^11^

#### Domestic physical activity

As domestic PA was only measured at adult time points (CDAH 1–3), trajectories spanning the entire life course were not possible. Two trajectories of domestic PA were observed across adulthood. These domestic PA trajectories did not significantly differ in their effect on cardiovascular outcomes. However, our observations of distinct patterns of adult domestic PA highlight the potential to increase total adult PA levels through interventions aimed at supporting those within the ‘persistently low’ domestic PA trajectory.

### Vascular function

We did not observe an association between trajectories of PA and measures of vascular function (Young’s elastic modulus and carotid distensibility). Existing cross-sectional studies suggest that total (mins/week) and light-to-moderate intensity PA may be associated with greater vascular function (lower stiffness) in older adults (>60 years), whereas vigorous PA may be necessary to elicit similar vascular benefits in younger and middle-aged populations.^41–43^ It is likely that our lack of PA intensity measures prevented our own observation of the association between PA and vascular function, given the age range of participants assessed. Further, as our participants have not yet reached older-adulthood, potential relationships between PA duration and vascular function (as previously suggested^41^) could not be examined. Future research incorporating objective measures of PA intensity and extended follow-up into older adulthood may provide greater insight into these complex relationships.

### Strengths and limitations

This study was strengthened by its use of a large, nationally representative cohort of children at baseline, with a long period of follow-up into mid-adulthood. Further, detailed PA measures were recorded in both childhood and adulthood. The use of LCGMM facilitated the first comparison of PA trajectories across all domains, adjusting for time-varying covariates. Identification of these trajectories highlights an opportunity to better understand the characteristics of individuals in which activity is persistently low, providing insights for domain-specific intervention development. LCGMM allowed for the *a posteriori* determination of PA trajectories using a data-driven approach. Unlike *a priori* classification, this data-driven technique reduces the risk of misclassification and information loss.^24^ It must be noted that with additional datapoints and larger samples, more granular trajectories might emerge than those of this study. These factors should be considered when interpreting the largely neutral vascular findings.

This study was limited by loss to follow-up. As those lost to follow-up often exhibit poorer health behaviours, it is possible that the findings of this study may underestimate the proportion of people who fall within the low PA trajectories and high-risk outcome categories. This study involved multiple comparisons across several correlated exposures and outcomes. To avoid inflating the risk of Type II error, formal correction for multiple testing was not applied. We acknowledge that this decision increases the potential for Type I error; therefore, we have interpreted findings with caution including focusing on effect sizes and confidence intervals rather than *P*-values. This study was limited as the use of a self-reported PA measurement in childhood and adulthood introduces the potential for recall error into this study. Specifically, the IPAQ-L does not capture bouts of activity under 10 min in duration potentially overlooking small bouts that may sum to provide greater totals.^44^ However, the IPAQ-L has been observed to have acceptable validity and high reliability in PA assessment.^45^ Childhood measures of PA presented in this study were assessed using different, unvalidated questions from those within the IPAQ-L used in adulthood, and therefore may not be directly aligned with adult activity domains^15^ (e.g. the potentially differing nature of compulsory and structured school-based and occupational activity). Further, the questions used in childhood were not validated, and as such, the effect of childhood PA on adult CVD and life course trajectories may be underestimated. We were unable to examine PA intensity across the life course due to the inconsistent availability of these data over the study period. Future research incorporating objective measures of intensity and extended follow-up into older adulthood is warranted. Finally, measures of carotid-to-femoral pulse wave velocity—a key marker of vascular degradation—were not available in this study.

## Conclusions

Maintaining consistently high levels of total and leisure-time activity across the life course is associated with more favourable vascular structure in mid-adulthood compared to lower levels of these types of activity. The early-life origins of vascular degradation, coupled with the distinct trajectories of PA identified across the life course, underscore the necessity of early and sustained PA for optimal vascular health. Negative relationships between school/occupational activity trajectories and vascular structure warrant future examination to determine mechanisms of effect. Further examination of PA intensity and vascular function beyond mid-adulthood is also recommended. While trajectories of high leisure-time PA from childhood through adulthood were identified to be of benefit to vascular structure, the observation of associations among trajectories of total PA strengthens the case that any PA may be beneficial.

## Supplementary Material

oeag038_Supplementary_Data

## Data Availability

Data is available upon reasonable request from the corresponding author. See here for further information: https://www.utas.edu.au/menzies/research/prevention-health-services-wellbeing/childhood-determinants-of-adult-health-study.

## Lead author biography

Jack T. Evans is a life course epidemiologist and cardiovascular researcher at the Menzies Institute for Medical Research, University of Tasmania, Australia. Dr Evans’ research aims understand how social and biological factors may affect health and behavioural outcomes. His primary interest is in the use of advanced statistics to predict and observe patterns of change from childhood to adulthood. His current work aims to develop a programme of precision cardiovascular disease prevention that supports the earlier identification of at-risk individuals and the formation of interventions (e.g., increasing physical activity and disease screening) that are optimized for specific populations and life periods.
