# Supplementary material for: Trajectories of total and domain-specific physical activity and vascular structure and function in mid-adulthood: the Childhood Determinants of Adult Health study
Source: Eur Heart J Open. 2026 Mar 18;6(2):oeag038. doi: 10.1093/ehjopen/oeag038 (PMC13061350; doi:10.1093/ehjopen/oeag038)
Supplement: oeag038_Supplementary_Data [file oeag038_supplementary_data.docx]

**SUPPLEMENTARY MATERIALS**

Jack T. Evans ^1^, Verity J. Cleland ^1,5,^*, Seana Gall ^1,2^, Terence Dwyer ^3,4^, Alison J. Venn ^1^, Rachel Climie ^1,6,^*

^1.^ Menzies Institute for Medical Research, University of Tasmania, Tasmania, Australia

^2.^ Adjunct Associate Professor, School of Clinical Sciences, Monash University, Australia

^3.^ Nuffield Department of Women’s & Reproductive Health, University of Oxford, United Kingdom

^4.^ Murdoch Children’s Research Institute, Melbourne, Australia

^5.^ Honorary Fellow, School of Exercise and Nutrition Sciences, Deakin University, Australia

^6.^ Université Paris Cité, INSERM, U970, Paris Cardiovascular Research Center (PARCC), Paris, France

* Joint senior authorship

**Supplementary Material 1 – STROBE Flowchart and Checklist**

**
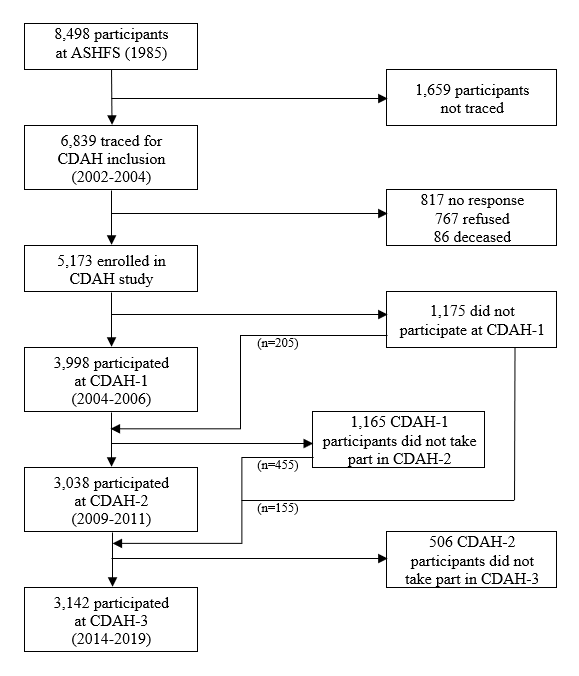
**

**Supplementary Figure 1.** Flowchart of participation in the Childhood Determinants of Adult Health Study

| **Supplementary Table 1.** STROBE Statement—Checklist of items that should be included in reports of *cohort studies* | | | |
| --- | --- | --- | --- |
|  | Item No | Recommendation | Page |
| **Title and abstract** | 1 | (*a*) Indicate the study’s design with a commonly used term in the title or the abstract | 1-2 |
|  |  | (*b*) Provide in the abstract an informative and balanced summary of what was done and what was found | 2 |
| Introduction | | |  |
| Background/rationale | 2 | Explain the scientific background and rationale for the investigation being reported | 3-4 |
| Objectives | 3 | State specific objectives, including any prespecified hypotheses | 4 |
| Methods | | |  |
| Study design | 4 | Present key elements of study design early in the paper | 5 |
| Setting | 5 | Describe the setting, locations, and relevant dates, including periods of recruitment, exposure, follow-up, and data collection | 5 |
| Participants | 6 | (*a*) Give the eligibility criteria, and the sources and methods of selection of participants. Describe methods of follow-up | 5-9 |
|  |  | (*b*) For matched studies, give matching criteria and number of exposed and unexposed | na |
| Variables | 7 | Clearly define all outcomes, exposures, predictors, potential confounders, and effect modifiers. Give diagnostic criteria, if applicable | 5-9  S5-S9 |
| Data sources/ measurement | 8* | For each variable of interest, give sources of data and details of methods of assessment (measurement). Describe comparability of assessment methods if there is more than one group | 5-9  S5-S9 |
| Bias | 9 | Describe any efforts to address potential sources of bias | 5-9  S5-S11 |
| Study size | 10 | Explain how the study size was arrived at | 5-10 |
| Quantitative variables | 11 | Explain how quantitative variables were handled in the analyses. If applicable, describe which groupings were chosen and why | 5-9  S5-S11 |

| **Supplementary Table 1.** *continued*. STROBE Statement—Checklist of items that should be included in reports of *cohort studies* | | | | |
| --- | --- | --- | --- | --- |
| Statistical methods | | 12 | (*a*) Describe all statistical methods, including those used to control for confounding | 7-9 |
|  |  |  | (*b*) Describe any methods used to examine subgroups and interactions |  |
|  |  |  | (*c*) Explain how missing data were addressed |  |
|  |  |  | (*d*) If applicable, explain how loss to follow-up was addressed |  |
|  |  |  | (*e*) Describe any sensitivity analyses | 10-11 |
| Results | | | |  |
| Participants | 13* | (a) Report numbers of individuals at each stage of study—e.g., numbers potentially eligible, examined for eligibility, confirmed eligible, included in the study, completing follow-up, and analysed | | 8  Fig.1 & S1 |
|  |  | (b) Give reasons for non-participation at each stage | |  |
|  |  | (c) Consider use of a flow diagram | |  |
| Descriptive data | 14* | (a) Give characteristics of study participants (e.g., demographic, clinical, social) and information on exposures and potential confounders | | 8  Tab.1 |
|  |  | (b) Indicate number of participants with missing data for each variable of interest | |  |
|  |  | (c) Summarise follow-up time (e.g., average and total amount) | |  |
| Outcome data | 15* | Report numbers of outcome events or summary measures over time | | 12  Tab.1  Fig.1 |
| Main results | 16 | (*a*) Give unadjusted estimates and, if applicable, confounder-adjusted estimates and their precision (eg, 95% confidence interval). Make clear which confounders were adjusted for and why they were included | | Tab.2-6  Tab.S2-S6 |
|  |  | (*b*) Report category boundaries when continuous variables were categorized | | 7, S5-S7 |
|  |  | (*c*) If relevant, consider translating estimates of relative risk into absolute risk for a meaningful time period | |  |
| Other analyses | 17 | Report other analyses done—e.g., analyses of subgroups and interactions, and sensitivity analyses | | S10-S18  Tab.S2-S6 |

**Supplementary Material 2 - Vascular Outcomes & Covariates**

**OUTCOMES**

The outcomes of carotid intima-media thickness, the presence of carotid plaques, Young’s Elastic Modulus, and carotid distensibility were measured in this study and are detailed as follows:

**Carotid ultrasound measurements**

Participants were assessed in the supine position using a SC2000 High-resolution ultrasound system (Siemens Healthcare, Mountainview, CA, USA) and a linear array transducer (Siemens 9L4) for all carotids image acquisitions. All carotid parameters were measured using semi-automated edge detection measurement software (Image-Arena M’ath, 4.6.3.9, TomTec Imaging Systems Edisonstr.6/85716 GmbH) and analysed offline blinded to the participant’s data by one single observer. To assess carotid artery elasticity indices, the best-quality cardiac cycle was selected from the 5-second image clips. The common carotid diameter 10 mm from carotid bifurcation was measured from the B-mode images with ultrasonic callipers at least twice in end diastole and end systole, respectively. The means of the measurements were used as the end-diastolic and end-systolic diameters. Ultrasound and brachial blood pressure measurements were used to calculate the following elasticity indices - where D_d_ is the intra-luminal diastolic diameter, D_s_ is the systolic diameter, P_s_ is systolic blood pressure, P_d_ is diastolic blood pressure, and IMT is carotid artery intima-media thickness.

Carotid intima-media thickness - Carotid intima–media thickness (cIMT) in adulthood was measured in the left common carotid far-wall using established protocols (1). Multiple 3- to 5-second real-time images were recorded that included the beginning of the carotid bulb and approximately 30 mm of the common carotid artery. From these images, the two highest-quality end-diastolic frames were selected by the reader for measurement. From each of these images, six measurements of the common carotid far wall were taken approximately 10 mm before the border of the carotid bulb to derive mean and maximum carotid IMT. Intra-rater reproducibility assessed with an average absolute difference (SD) of 0.02 (±0.04) mm determined. High-risk cIMT was defined as values equal to or greater than the 90th percentile (2).

Carotid plaques

Plaques were identified as any focal structure encroaching into the arterial lumen by ≥0.5 mm or 50% of the surrounding intima-media thickness value, or demonstrating a thickness >1.5 mm as measured from the intima-lumen interface to the media-adventitia interface (3).

Young's [elastic modulus](https://www.sciencedirect.com/topics/nursing-and-health-professions/young-modulus) **–** YEM (mmHg.mm) gives an estimate of arterial stiffness that is independent of wall (intima-media) thickness and was calculated as follows: YEM=([P_s_−P_d_]×D_d_)/([D_s_−D_d_]/IMT). High-risk YEM was defined as values equal to or greater than the 90th percentile

Carotid distensibility

Carotid distensibility (CD; %/10mmHg), a measure of carotid artery elasticity, was determined as follows: CD = (D_s_ – D_d_]/D_d_)/(brachial SBP− brachial DBP) (4). High-risk cD was defined as values equal to or greater than the 90th percentile

**COVARIATES**

Age - At baseline, date of birth was reported via questionnaire, years of age was then calculated at each follow-up.

Sex - Biological sex were reported via questionnaire at ASHFS baseline.

Body mass index - In childhood, height was assessed using a KaWe height tape (KaWe Kirchner & Wilhelm, Asperg, Germany); in adulthood a Leicester height measure (Invicta, Leicester, UK) was used. Childhood body mass was measured via beam or medical spring scales at baseline; adult body mass was assessed using Heine scales (Heine, Dover, NH) at CDAH-1 and CDAH-3 follow-ups. Height and body mass were self-reported at CDAH-2. Body mass index (BMI) (kg/m^2^) was then calculated as body mass (kg) divided by height (m) squared.

High-density lipoprotein (HDL) and Low-density lipoprotein (LDL) –

Fasting blood samples were taken from the antecubital fossa, low-density lipoprotein (LDL) and high-density lipoprotein (HDL) cholesterol levels were measured (mmol/l).

Smoking status - Childhood smoking status was categorised by duration: “I don’t smoke”, “just started”, “1-12 months”, and “>1 year” at baseline. Adult smoking was reported by frequency: “never smoked”, “ex-smoker”, “less than weekly” “weekly”, and “daily” at each CDAH follow-up.

Occupation - Occupation was self-reported at each adult CDAH follow-up and classified into the following groups “manager/professionals”, “white collar (clerical, sales, service)”, “blue collar (trades, labour)”, and not in labour force (students, unemployed, retired, home duties).

Self-rated health - At each timepoint self-rated health was reported as “poor”, “fair”, “good”, “very good”, and “excellent”.

Education - At baseline, participant education level was recorded as scholastic ability indicated by the student’s teacher on a scale ranging from 1 – 5 (i.e., “excellent” to “poor”).

At CDAH adult follow-ups, the participants highest level of education was recorded via questionnaire and categorised as: low (primary and high school education only), medium (undergraduate diploma, associate diploma, skilled and basic vocational training), and high (higher degree, post graduate diploma, bachelor’s degree).

Marital status - During adulthood, marital status was reported via questionnaire and categorised as “single”, “married/living as married relationships”, “divorced/separated”, and “widowed”.

**Supplementary Material 3 – Latent Class Approach**

Latent Class Growth Mixture Modelling (LCGMM), a form of trajectory analysis, was performed to assess total physical activity and each of the four comprising domains (leisure, occupational, transport, and domestic) across the adult life-period. Traditional longitudinal methodologies assume that every participant follows a similar trajectory, this single average trajectory is used to define the population and covariates are then used to explain variability. As such, individual deviations from mean population longitudinal trends are represented in terms of individual-level random effects, indicating how individual parameters of change differ from those of the population-average. These oversimplifications result in a single homogeneous population-average estimate of variance. LCGMM overcomes these limitations by considering that a population consists of several heterogeneous subgroups, each with its own distinct patterns (5). Data is used to infer the number of distinct trajectories/classes, the unique shape of each class, and the number of individuals probabilistically assigned to each class (class membership). The use of this data driven approach allows LCGMM to overcome potential information loss and issues of misclassification that are otherwise experienced when subjectively clustering participants into previously defined groups (5). Through the integration of residual adjustment into the LCGMM technique, this trajectory analysis can adjust for time-varying covariates (6); a crucial requirement for the analysis of physical activity trajectories where covariates influence the repeated measures of physical activity at each timepoint. Latent class analysis was performed in the following stages:

*Adjustment for time-varying covariates*

Previous literature indicates that individual-level correlates influence each of the differing physical activity domains in adulthood (7, 8). To adjust for these time-varying covariates, the LCGMM process (6, 9) was undertaken using a residual adjustment approach (6). First, residuals were extracted for each domain of activity using linear regression, theoretically representing the effect unexplained by the relationship between the exposures (covariates) and physical activity levels at each timepoints. That is, a measure of the activity undertaken independent of covariates. Only participants with observations of both covariates and physical activity at each timepoint had residuals (adjusted physical activity) extracted.

*Lifecourse trajectories of total and domain specific physical activity.*

Trajectory analysis of total physical activity and each comprising domain (leisure, occupational, and transport) was performed across the lifecourse to determine whether distinct trajectories existed among this Australian population. The domain of domestic physical activity was assessed across adult timepoints only due to a lack of childhood assessment. LCGMM was performed among participants with observations from a minimum of two out of the four (including ASHFS) timepoints.

*Model fit*

A series of models considering linear, quadratic, and cubic polynomial specifications were fit as a function of timepoint using residuals of total physical activity and each individual domain. Model building and class enumeration was performed in line with recommendations of Proust-Lima (10). The model with the best fit was selected based on goodness of fit, Akaike information criteria (AIC), Bayesian information criteria (BIC) indices, and the proportion of participants with a mean posterior class membership probability >0.7.

**Supplementary Material 4 – Supplementary Results**

| **Supplementary Table 2.** Physical activity of Australian CDAH Study participants within each latent total and domain specific trajectory | | | | | | | | | | | | | | | | | | | | | | | | |
| --- | --- | --- | --- | --- | --- | --- | --- | --- | --- | --- | --- | --- | --- | --- | --- | --- | --- | --- | --- | --- | --- | --- | --- | --- |
| Domain | Total | | | Leisure | | | | | | | | | | Transport | | | Occupational | | | Domestic | | | | |
| Trajectory | Low | Low to High | High | | Low | | | High/Decreasing | | | High | | | Low | | High | Low | Low to High | High | | Low | | High | |
| % (N) | 21.6 (498) | 73.5 (1699) | 4.9 (114) | | 85.5 (1975) | | | 2.2 (51) | | | 12.3 (285) | | | 87.7 (2026) | | 12.3 (285) | 83.4 (1928) | 13.1 (303) | 3.5 (80) | | 78.8 (1821) | | 21.2 (490) | |
| **Total PA (min/week)** | | | | | | | | | | | | | | | | | | | | | | | | |
| ASHFS | 310.0  (185, 515) | 330.0 (190.0,542.0) | 1381.5  (470, 1980) | | 300.0  (180, 468) | | | 1760.0  (1305, 2230) | | | 780.0  (420, 1070) | | | 320.0  (190, 540) | | 358.0  (210, 680) | 320.0  (190, 540) | 315.0  (180, 540) | 450.0  (275, 1089) | | 320.0  (190, 540) | | 330.0  (195, 580) | |
| CDAH-1 | 545.0  (310, 851) | 1169.1  (750, 1499) | 1591.5  (969, 2021) | | 637.6  (360, 1024) | | | 1058.7  (730, 1420) | | | 780.0  (494, 1228) | | | 630.0  (350, 1000) | | 1045.4  (637, 1518) | 598.3  (330, 921) | 1061.8  (725, 1390) | 1803.6  (1417, 2100) | | 580.0  (325, 945) | | 1018.8  (695, 1359) | |
| CDAH-2 | 522.0  (313, 790) | 1291.7  (1012, 1618) | 1458.8  (778, 1999) | | 646.1  (360, 1020) | | | 990.0  (660, 1546) | | | 930.8  (570, 1350) | | | 630.0  (360, 995) | | 1076.5  (767, 1682) | 600.0  (350, 960) | 1158.8  (776, 1476) | 1135.4  (690.9, 1817) | | 555.0  (330, 879) | | 1133.4  (840, 1560) | |
| CDAH-3 | 560.0  (330, 810) | 1140.0  1182, 1779) | 1145  (471, 1698) | | 650.0  (380, 1021) | | | 911.4  (355, 1290) | | | 1047.4  (667, 1410) | | | 640.0  (370, 1003) | | 1228.4  (819, 1652) | 620.0  (360, 945) | 1381.5  (1080, 1772) | 919.0  (471, 1672) | | 620.0  (360, 986) | | 1031.5  (702, 1505) | |
| **Leisure PA (min/week)** | | | | | | | | | | | | | | | | | | | | | | | | |
| ASHFS | 120.0  (10, 270) | 132.5  (30, 330) | 720.0  (120, 1500) | | 115.0  (0,240) | | | 1500.0  (1180, 2040) | | | 570.0  (210, 870) | | | 120.0  (25, 300) | | 120.0  (20, 360) | 120.0  (24, 300) | 145.0  (30, 320) | 150.0  (0, 360) | | | 125.0  (20, 300) | | 120.0  (30, 350) |
| CDAH-1 | 90.0  (0,210) | 150.0  (38, 321) | 133.5  (45, 315) | | 90.0  (0, 204) | | | 207.9  (60, 864) | | | 252.5  (90, 450) | | | 92.4  (0,225) | | 120.0  (30, 300) | 100.1  (14, 240) | 92.1  (0, 236) | 90.5  (0, 236) | | | 100.0  (0, 240) | | 103.3  (0, 220) |
| CDAH-2 | 90.0  (8, 205) | 179.9  (40, 336) | 226.4  (85, 420) | | 100.0  (5, 210) | | | 240.0  (151, 470) | | | 263.8  (90, 530) | | | 105.0  (20, 230) | | 175.4  (51, 315) | 120.0  (22, 240) | 82.0  (0, 180) | 100.0  (19, 242) | | | 115.2  (20, 240) | | 122.5  (20, 267) |
| CDAH-3 | 120.0  (30, 240) | 218.0  (51, 396) | 105.0  (30, 385) | | 12.0  (30, 240) | | | 225.0  (45, 690) | | | 299.1  (60, 600) | | | 120.0  (30, 270) | | 171.9  (60, 309) | 140.0  (30, 280) | 95.9  (26, 230) | 88.2  (0, 240) | | | 135.0  (30, 270) | | 120.0  (30, 283) |
| **Transport PA (min/week)** | | | | | | | | | | | | | | | | | | | | | | | | |
| ASHFS | 20.0  (0, 75) | 15.0  (0, 75) | 32.5  (0, 100) | | 20.0  (0, 75) | | 25.0  (0, 80) | | | 26.0  (0, 80) | | | 20.0  (0, 70) | | 40.0  (0, 100) | | 20.0  (0, 75) | 5.0  (0, 55) | 40.0  (0, 77) | | | 20.0  (0, 75) | | 20.0  (0, 75) |
| CDAH-1 | 34.3  (0, 120) | 80.0  (0, 210) | 120.0  (36, 265) | | 41.2  (0, 140) | | 60.0  (0, 150) | | | 70.0  (0, 210) | | | 36.0  (0, 120) | | 235.0  (75, 420) | | 50.0  (0, 150) | 40.0  (0, 120) | 75.0  (0, 180) | | | 45.0  (0, 140) | | 54.7  (0, 140) |
| CDAH-2 | 40.0  (0, 120) | 100.0  (0, 273) | 120.0  (0, 260) | | 45.0  (0, 140) | | 75.0  (0, 160) | | | 75.0  (0, 180) | | | 40.0  (0, 120) | | 360.0  (140, 490) | | 50.0  (0, 150) | 40.0  (0, 120) | 83.5  (0, 210) | | | 45.0  (0, 140) | | 50.0  (0, 180) |
| CDAH-3 | 45.0  (0, 135) | 150.0  (25, 400) | 60.0  (0, 252) | | 60.0  (0, 160) | | 40.0  (0, 150) | | | 60.0  (0, 210) | | | 41.4  (0, 120) | | 420.0  (315, 616) | | 60.0  (0, 175) | 40.0  (0, 180) | 40.0  (0, 140) | | | 60.0  (0, 160) | | 60.0  (0, 226) |
| **Occupational PA (min/week)** | | | | | | | | | | | | | | | | | | | | | | | | |
| ASHFS | 110.0  (60, 180) | 110.0  (60, 180) | 167.5  (100, 280) | 110.0  (60, 180) | | 130.0  (60, 190) | | | 120.0  (60, 173) | | | 115.0  (60, 180) | | | 120.0  (60, 180) | | 115.0  (60, 180) | 110.0  (60, 180) | 180.0  (90, 475) | | | 113.0  (60, 180) | | 120.0  (60, 190) |
| CDAH-1 | 0.0  (0.0, 265) | 274.6  (0, 609) | 424.3  (41, 1106) | 20.0  (0, 399) | | 90.0  (0, 510) | | | 2.5  (0, 378) | | | 15.0  (0, 371) | | | 149.1  (0, 522) | | 0.0  (0, 220) | 540.0  (200, 852) | 1267.5  (1012, 1541) | | | 28.8  (0, 416) | | 0.0  (0, 351) |
| CDAH-2 | 0.0  (0, 129) | 270.8  (0, 620) | 305.0  (0, 629) | 0.0  (0, 279) | | 310.8  (0, 540) | | | 7.2  (0, 303) | | | 0.0  (0, 270) | | | 45.0  (0, 360) | | 0.0  (0, 120) | 613.2  (310, 910) | 699.9  (180, 1326) | | | 0.0  (0, 286) | | 0.0  (0, 301) |
| CDAH-3 | 0.0  (0, 186) | 384.9  (0, 843) | 10.0  (0, 706) | 20.0  (0, 334) | | 0.0  (0, 360) | | | 10.0  (0, 352) | | | 17.5  (0, 330) | | | 0.0  (0, 372) | | 0.0  (0, 180) | 874.3  (659, 1208) | 56.5  (0, 920) | | | 17.5  (0, 348) | | 15.0  (0, 337) |
| **Domestic PA (min/week)** | | | | | | | | | | | | | | | | | | | | | | | | |
| ASHFS | - | - | - | - | | - | | | - | | | - | | | - | | - | - | - | | | - | | - |
| CDAH-1 | 140.0  (50, 300) | 265.9  (116, 600) | 335.8  (120, 635) | 160.0  (60, 355) | | 144.0  (120, 480) | | | 171.1  (56, 360) | | | 157.5  (60, 355) | | | 180.0  (90, 360) | | 162.7  (60, 360) | 180.0  (77, 360) | 134.0  (25, 329) | | | 120.0  (45, 240) | | 562.4  (314, 874) |
| CDAH-2 | 166.1  (75, 337) | 402.5  (170, 744) | 480.0  (240, 738) | 200.0  (90, 432) | | 210.0  (120, 480) | | | 231.2  (110, 495) | | | 196.4  (90, 420) | | | 270.9  (129, 648) | | 200.0  (90, 450) | 235.7  (120, 420) | 121.2  (63, 317) | | | 150.0  (70, 283) | | 707.4  (443, 920) |
| CDAH-3 | 150.0  (60, 277) | 360.0  (180, 720) | 270.0  (120, 612) | 180.0  (75, 360) | | 180.0  (20.0, 320) | | | 190.0  (85, 402) | | | 172.4  (72, 336) | | | 264.1  (120, 540) | | 180.0  (75, 360) | 180.0  (90, 360) | 156.5  (70, 283) | | | 144.7  (60, 260) | | 570.0  (266, 840) |
| Data presented as median (inter-quartile range). ASHFS = Australian Schools Health and Fitness Survey, CDAH = Childhood Determinants of Adult Health Study | | | | | | | | | | | | | | | | | | | | | | | | |

| **Supplementary Table 3.** Associations between transport-related physical activity trajectories and dichotomous adult vascular structure and function outcomes. | | | | |
| --- | --- | --- | --- | --- |
| Outcome,  Latent transport-related physical activity trajectory | Model 1 | | Model 2 | |
|  | n / N | RR (95% CI) | n / N | RR (95% CI) |
| High-risk Young’s Elastic Modulus | 79 / 765 |  | 76 / 713 |  |
| Persistently low |  | REF |  | REF |
| High and increasing |  | 1.676 (0.861, 2.492) |  | 1.576 (0.835, 2.317) |
| High-risk carotid distensibility | 75/765 |  | 72/713 |  |
| Persistently low |  | REF |  | REF |
| High and increasing |  | 1.041 (0.430, 1.652) |  | 1.127 (0.452, 1.803) |
| High-risk cIMT | 104 / 914 |  | 97 / 853 |  |
| Persistently low |  | REF |  | REF |
| High and increasing |  | 1.066 (0.538, 1.595) |  | 1.053 (0.501, 1.606) |
| Presence of carotid plaques | 116 / 867 |  | 110 / 807 |  |
| Persistently low |  | REF |  | REF |
| High and increasing |  | 1.016 (0.523, 1.510) |  | 0.989 (0.495, 1.484) |
| n= number with high-risk outcomes; N= number with outcomes assessed; RR= relative risk; CI= confidence interval; cIMT= carotid intima-media thickness  Model 1 = adjusted for age, and sex, and leisure, occupational, and domestic physical activity.  Model 2 = additionally adjusted for occupation-type, body mass index, smoking status, high-density lipoprotein, low-density lipoprotein, and area-level socio-economic status. | | | | |

| **Supplementary Table 4.** Associations between occupational physical activity trajectories and dichotomous adult vascular structure and function outcomes. | | | | |
| --- | --- | --- | --- | --- |
| Outcome,  Latent occupational physical  activity trajectory | Model 1 | | Model 2 | |
|  | n / N | RR (95% CI) | n / N | RR (95% CI) |
| High-risk Young’s Elastic Modulus | 79 / 765 |  | 76 / 713 |  |
| Persistently low |  | REF |  | REF |
| Low to high |  | 1.261 (0.567, 1.954) |  | 1.142 (0.580, 1.705) |
| High and increasing |  | 0.583 (-0.507, 1.673) |  | 0.699 (-0.608, 2.005) |
| High-risk carotid distensibility | 75/765 |  | 72/713 |  |
| Persistently low |  | REF |  | REF |
| Low to high |  | 1.350 (0.605, 2.095) |  | 1.371 (0.600, 2.142) |
| High and increasing |  | 0.572 (-0.504, 1.648) |  | 0.635 (-0.562, 1.833) |
| High-risk cIMT | 104 / 914 |  | 97 / 853 |  |
| Persistently low |  | REF |  | REF |
| Low to high |  | 0.746 (0.298, 1.203) |  | 0.657 (0.199, 1.111) |
| High and increasing |  | 1.645 (0.426, 2.864) |  | 1.742 (0.471, 3.012) |
| Presence of carotid plaques | 116 / 867 |  | 110 / 807 |  |
| Persistently low |  | REF |  | REF |
| Low to high |  | 1.152 (0.613, 1.692) |  | 1.174 (0.597, 1.752) |
| High and increasing |  | 0.842 (-0.261, 1.946) |  | 1.085 (-0.341, 1.512) |
| n= number with high-risk outcomes; N= number with outcomes assessed; RR= relative risk; CI= confidence interval; cIMT= carotid intima-media thickness  Model 1 = adjusted for age, and sex, and leisure, transport, and domestic physical activity.  Model 2 = additionally adjusted for occupation-type, body mass index, smoking status, high-density lipoprotein, low-density lipoprotein, and area-level socio-economic status. | | | | |

| **Supplementary Table 5.** Associations between domestic physical activity trajectories and dichotomous adult vascular structure and function outcomes. | | | | |
| --- | --- | --- | --- | --- |
| Outcome,  Latent domestic physical  activity trajectory | Model 1 | | Model 2 | |
|  | n / N | RR (95% CI) | n / N | RR (95% CI) |
| High-risk Young’s Elastic Modulus |  |  |  |  |
| Persistently low |  | REF |  | REF |
| Consistently high |  | 1.676 (0.861, 2.492) |  | 1.576 (0.835, 2.317) |
| High-risk carotid distensibility | 75/765 |  | 72/713 |  |
| Persistently low |  | REF |  | REF |
| Consistently high |  | 1.041 (0.430, 1.652) |  | 1.127 (0.452, 1.803) |
| High-risk cIMT | 104 / 914 |  | 97 / 853 |  |
| Persistently low |  | REF |  | REF |
| Consistently high |  | 0.669 (0.297, 1.040) |  | 0.669 (0.292, 1.047) |
| Carotid Plaques | 116 / 867 |  | 110 / 807 |  |
| Persistently low |  | REF |  | REF |
| Consistently high |  | 1.421 (0.868, 1.975) |  | 1.462 (0.894, 2.030) |
| n= number with high-risk outcomes; N= number with outcomes assessed; RR= relative risk; CI= confidence interval; cIMT= carotid intima-media thickness  Model 1 = adjusted for age, and sex, and leisure, transport, and occupational physical activity..  Model 2 = additionally adjusted for occupation-type, body mass index, smoking status, high-density lipoprotein, low-density lipoprotein, and area-level socio-economic status. | | | | |

| **Supplementary Table 6.** Associations between total physical activity trajectories and continuous adult vascular structure and function outcomes. | | | | |
| --- | --- | --- | --- | --- |
| Outcome,  Latent total physical activity trajectory | Model 1 | | Model 2 | |
|  |  | β (95% CI) |  | β (95% CI) |
| Carotid intima-media thickness (mm) | 914 |  | 853 |  |
| Persistently low |  | REF |  | REF |
| Low to high |  | **0.017 (0.001, 0.033)** |  | **0.020 (0.003, 0.036)** |
| Consistently high |  | -0.009 (-0.045, 0.027) |  | -0.011 (-0.047, 0.025) |
| Young’s Elastic Modulus (mmHg.mm) | 765 |  | 713 |  |
| Persistently low |  | REF |  | REF |
| Low to high |  | 19.73 (-2.89, 42.35) |  | 16.91 (-5.99, 39.81) |
| Consistently high |  | 5.96 (-45.41, 57.33) |  | 7.97 (-44.07, 60.01) |
| Carotid distensibility (%/10mmHg) | 765 |  | 713 |  |
| Persistently low |  | REF |  | REF |
| Low to high |  | -0.55 (-0.014, 0.03) |  | -0.04 (-0.13, 0.05) |
| Consistently high |  | 0.00 (-0.20, 0.20) |  | -0.02 (-0.23, 0.18) |
| N = number of participants, 95%CI = 95% confidence interval  Model 1 = adjusted for age and sex.  Model 2 = additionally adjusted for occupation-type, body mass index, smoking status, high-density lipoprotein, low-density lipoprotein, and index of relative socio-economic advantage and disadvantage. | | | | |

| **Supplementary Table 7.** Associations between total physical activity trajectories among participants with complete case adult vascular structure and function outcomes | | | | |
| --- | --- | --- | --- | --- |
| Outcome,  Latent total physical activity trajectory | Dichotomous | | Continuous | |
|  | n / N | RR (95% CI) | N | β (95% CI) |
| Young’s Elastic Modulus  (High-risk \| mmHg.mm) | 79 / 769 |  | 769 |  |
| Persistently low | 20 / 166 | REF | 166 | REF |
| Low to high | 55 / 577 | 0.714 (0.388, 1.040) | 577 | -20.69 (-43.58, 2.21) |
| Consistently high | 4 / 26 | 1.132 (0.067, 2.198) | 26 | -18.90 (-73.70, 35.90) |
| Carotid distensibility  (High-risk \| %/10mmHg) | 75/ 769 |  | 769 |  |
| Persistently low | 14 / 166 | REF | 166 | REF |
| Low to high | 56 / 577 | 1.06 (0.488, 1.632) | 577 | 0.06 (-0.03, 0.14) |
| Consistently high | 5 / 26 | 2.072 (0.257, 3.888) | 26 | 0.08 (-0.12, 0.28) |
| Carotid IMT  (High-risk \| mm) | 91/ 769 |  | 769 |  |
| Persistently low | 20 / 166 | REF | 166 | REF |
| Low to high | 68 / 577 | 0.906 (0.494, 1.319) | 577 | -0.01 (-0.03, 0.01) |
| Consistently high | 3 / 26 | 0.838 (-0.099, 1.775) | 26 | -0.02 (-0.07, 0.02) |
| Presence of carotid plaques | 107/ 769 |  |  |  |
| Persistently low | 23 / 166 | REF |  |  |
| Low to high | 79 / 577 | 0.937 (0.534, 1.339) |  |  |
| Consistently high | 5 / 26 | 1.282 (0.170, 2.393) |  |  |
| n= number with high-risk outcomes; N= number with outcomes assessed; RR= relative risk; CI= confidence interval; IMT= intima-media thickness  Models adjusted for age and sex. | | | | |

| **Supplementary Table 8.** Associations between occupational physical activity trajectories and continuous adult vascular structure and function outcomes. | | | | |
| --- | --- | --- | --- | --- |
| Outcome,  Latent occupational physical activity trajectory | Model 1 | | Model 2 | |
|  |  | β (95% CI) |  | β (95% CI) |
| Carotid intima-media thickness (mm) | 914 |  | 853 |  |
| Persistently low |  | REF |  | REF |
| Low to high |  | -0.012 (-0.032, 0.007) |  | -0.013 (-0.032, 0.007)) |
| High and increasing |  | **0.056 (0.003, 0.110)** |  | **0.057 (0.004, 0.111)** |
| Young’s Elastic Modulus (mmHg.mm) | 765 |  | 713 |  |
| Persistently low |  | REF |  | REF |
| Low to high |  | 15.74 (-11.94, 43.43) |  | 8.44 (-19.08, 35.97) |
| High and increasing |  | 6.34 (-62.42, 75.10) |  | 4.220 (-65.60, 74.01) |
| Carotid distensibility (%/10mmHg) | 765 |  | 713 |  |
| Persistently low |  | REF |  | REF |
| Low to high |  | -0.07 (-0.17, 0.03) |  | -0.05 (-0.16, 0.05) |
| High and increasing |  | 0.123 (-0.15, 0.40) |  | 0.14 (-0.15, 0.43) |
| N = number of participants, 95%CI = 95% confidence interval  Model 1 = adjusted for age, sex, and leisure, transport, and domestic physical activity  Model 2 = additionally adjusted for occupation-type, body mass index, smoking status, high-density lipoprotein, low-density lipoprotein, and index of relative socio-economic advantage and disadvantage. | | | | |

| **Supplementary Table 9.** Associations between occupational physical activity trajectories among participants with complete case adult vascular structure and function outcomes | | | | |
| --- | --- | --- | --- | --- |
| Outcome,  Latent occupational physical  activity trajectory | Dichotomous | | Continuous | |
|  | n / N | RR (95% CI) | N | β (95% CI) |
| Young’s Elastic Modulus  (High-risk \| mmHg.mm) | 79 / 769 |  | 769 |  |
| Persistently low | 66 / 656 | REF | 656 | REF |
| Low to high | 12 / 99 | 1.264 (0.568, 1.959) | 99 | 16.03 (-11.91, 43.98) |
| High and increasing | 1 / 14 | 0.582 (0.506, 1.671) | 14 | 6.07 (-63.26, 75.41) |
| Carotid distensibility  (High-risk \| %/10mmHg) | 75 / 769 |  | 769 |  |
| Persistently low | 69 / 656 | REF | 656 | REF |
| Low to high | 12 / 99 | 1.353 (0.606, 2.099) | 99 | -0.07 (-0.17, 0.03) |
| High and increasing | 1 / 14 | 0.567 (0.500, 1.633) | 14 | 0.31 (-0.15, 0.40) |
| Carotid IMT  (High-risk \| mm) | 91 / 769 |  | 769 |  |
| Persistently low | 79 / 656 | REF | 656 | REF |
| Low to high | 8 / 99 | 0.707 (0.224, 1.191) | 99 | -0.01 (-0.04, 0.01) |
| High and increasing | 4 / 14 | 1.557 (0.242, 2.872) | 14 | 0.06 (-0.01, 0.12) |
| Presence of carotid plaques | 107 / 769 |  |  |  |
| Persistently low | 89 / 656 | REF |  |  |
| Low to high | 16 / 99 | 1.212 (0.362, 1.793) |  |  |
| High and increasing | 2 / 14 | 0.921 (0.276, 2.119) |  |  |
| n= number with high-risk outcomes; N= number with outcomes assessed; RR= relative risk; CI= confidence interval; IMT= intima-media thickness  Models adjusted for age, sex, and leisure, transport, and domestic physical activity | | | | |

| **Supplementary Table 10.** Associations between leisure physical activity trajectories among participants with complete case adult vascular structure and function outcomes | | | | |
| --- | --- | --- | --- | --- |
| Outcome,  Latent leisure-time physical activity trajectory | Dichotomous | | Continuous | |
|  | n / N | RR (95% CI) | N | β (95% CI) |
| Young’s Elastic Modulus  (High-risk \| mmHg.mm) | 79 / 769 |  | 769 |  |
| Persistently low | 65 / 652 | REF | 652 | REF |
| Low to high | 0 / 9 | NA | 9 | -4.63 (-90.00, 80.75) |
| Consistently high | 14 / 108 | 1.118 (0.539, 1.698) | 108 | -3.74 (-30.24, 22.76) |
| Carotid distensibility  (High-risk \| %/10mmHg) | 75 / 769 |  | 769 |  |
| Persistently low | 62 / 652 | REF | 652 | REF |
| Low to high | 1 / 9 | 0.871 (0.071, 2.458) | 9 | -0.04 (-0.36, 0.28) |
| Consistently high | 12 / 108 | 1.018 (0.446, 1.589) | 108 | 0.03 (-0.07, 0.13) |
| Carotid IMT  (High-risk \| mm) | 91 / 769 |  | 769 |  |
| Persistently low | 79 / 652 | REF | 652 | REF |
| Low to high | 3 / 9 | 2.040 (0.186, 3.894) | 9 | -0.04 (-0.10, 0.03) |
| Consistently high | 9 / 108 | 0.680 (0.237, 1.123) | 108 | -0.01 (-0.03, 0.01) |
| Presence of carotid plaques | 91 / 769 |  |  |  |
| Persistently low | 97 / 652 | REF |  |  |
| Low to high | 2 / 9 | 1.254 (0.288, 2.797) |  |  |
| Consistently high | 8 / 108 | **0.457 (0.142, 0.773)** |  |  |
| n= number with high-risk outcomes; N= number with outcomes assessed; RR= relative risk; CI= confidence interval; IMT= intima-media thickness  Models adjusted for age, sex, and transport, occupational, and domestic physical activity | | | | |

**Supplementary Material 5 – Supplementary Results (not cited in-text)**

TOTAL PHYSICAL ACTIVTY

| **Supplementary Table 11.** Associations between total physical activity trajectories and function outcomes among participants with complete case physical activity measures and adult vascular structure | | | | |
| --- | --- | --- | --- | --- |
| Outcome,  Latent total physical activity trajectory | Dichotomous | | Continuous | |
|  | n / N | RR (95% CI) | N | β (95% CI) |
| Young’s Elastic Modulus  (High-risk \| mmHg.mm) | 37 / 382 |  | 382 |  |
| Persistently low | 5 / 79 | REF | 329 | REF |
| Low to high | 31 / 295 | 1.447 (0.154, 2.740) | 48 | 2.28 (-29.79, 34.35) |
| Consistently high | 1 / 8 | 1.521 (1.377, 4.420) | 5 | -64.06 (-150.00, 21.85) |
| Carotid distensibility  (High-risk \| %/10mmHg) | 35 / 382 |  | 382 |  |
| Persistently low | 7 / 79 | REF | 329 | REF |
| Low to high | 27 / 295 | 0.867 (0.209, 1.525) | 48 | 0.01 (-0.12, 0.13) |
| Consistently high | 1 / 8 | 1.042 (-0.832, 2.915) | 5 | 0.25 (-0.15, 0.65) |
| Carotid IMT  (High-risk \| mm) | 47 / 477 |  | 477 |  |
| Persistently low | 10 / 99 | REF | 408 | REF |
| Low to high | 36 / 366 | 0.963 (0.339, 1.589) | 62 | -0.001 (-0.02, 0.02) |
| Consistently high | 1 / 12 | 0.933 (-0.866, 2.732) | 7 | -0.001 (-0.07, 0.04) |
| Presence of carotid plaques | 47 / 432 |  |  |  |
| Persistently low | 9 / 90 | REF |  |  |
| Low to high | 37 / 330 | 1.096 (0.344, 1.848) |  |  |
| Consistently high | 1 / 12 | 0.857 (-0.837, 2.551) |  |  |
| n= number with high-risk outcomes; N= number with outcomes assessed; RR= relative risk; CI= confidence interval; IMT= intima-media thickness  Models adjusted for age and sex. | | | | |

LEISURE TIME PHYSICAL ACTIVITY

| **Supplementary Table 12.** Associations between leisure-time physical activity trajectories and continuous adult vascular structure and function outcomes. | | | | |
| --- | --- | --- | --- | --- |
| Outcome,  Latent leisure-time physical activity trajectory | Model 1 | | Model 2 | |
|  |  | β (95% CI) |  | β (95% CI) |
| Carotid intima-media thickness (mm) | 914 |  | 853 |  |
| Persistently low |  | REF |  | REF |
| High and decreasing |  | -0.025 (-0.078, 0.027) |  | -0.009 (-0.066, 0.048) |
| Consistently high |  | 0.006 (-0.013, 0.025) |  | 0.004 (-0.016, 0.023) |
| Young’s Elastic Modulus (mmHg.mm) | 765 |  | 713 |  |
| Persistently low |  | REF |  | REF |
| High and decreasing |  | -21.08 (-95.69, 53.54) |  | 9.67 (-74.52, 93.86) |
| Consistently high |  | 4.14 (-22.56, 30.85) |  | 7.23 (-19.40, 33.86) |
| Carotid distensibility (%/10mmHg) | 765 |  | 713 |  |
| Persistently low |  | REF |  | REF |
| High and decreasing |  | 0.03 (-0.27, 0.33) |  | -0.02 (-0.21, 0.16) |
| Consistently high |  | 0.00 (-0.10, 0.10) |  | -0.00 (-0.06, 0.05) |
| N = number of participants, 95%CI = 95% confidence interval  Model 1 = adjusted for age, sex, and transport, occupational, and domestic physical activity  Model 2 = additionally adjusted for occupation-type, body mass index, smoking status, high-density lipoprotein, low-density lipoprotein, and index of relative socio-economic advantage and disadvantage. | | | | |

| **Supplementary Table 13.** Associations between leisure physical activity trajectories and adult vascular structure and function outcomes among participants with complete case physical activity measures | | | | |
| --- | --- | --- | --- | --- |
| Outcome,  Latent leisure-time physical activity trajectory | Dichotomous | | Continuous | |
|  | n / N | RR (95% CI) | N | β (95% CI) |
| Young’s Elastic Modulus  (High-risk \| mmHg.mm) | 37 / 382 |  | 382 |  |
| Persistently low | 33 / 329 | REF | 329 | REF |
| Low to high | 1 / 4 | NA | 48 | 58.11 (-140.00, 256.23) |
| Consistently high | 5 / 60 | 0.651 (0.027, 1.275) | 5 | -20.92 (-58.68, 16.83) |
| Carotid distensibility  (High-risk \| %/10mmHg) | 35 / 382 |  | 382 |  |
| Persistently low | 30 / 329 | REF | 329 | REF |
| Low to high | 0 / 2 | NA | 48 | -0.24 (-0.87, 0.40) |
| Consistently high | 5 / 51 | 0.851 (0.148, 1.554) | 5 | -0.13 (-0.03, 0.28) |
| Carotid IMT  (High-risk \| mm) | 47 / 477 |  | 477 |  |
| Persistently low | 41 / 411 | REF | 408 | REF |
| Low to high | 1 / 3 | 5.296 (-3.521, 14.112) | 62 | 0.04 (-0.07, 0.16) |
| Consistently high | 5 / 51 | 0.732 (0.950, 1.368) | 7 | 0.01 (-0.02, 0.04) |
| Presence of carotid plaques | 47 / 432 |  |  |  |
| Persistently low | 41 / 368 | REF |  |  |
| Low to high | 1 / 4 | 2.769 (-0.212, 7.661) |  |  |
| Consistently high | 5 / 60 | 0.685 (0.0.78, 1.292) |  |  |
| n= number with high-risk outcomes; N= number with outcomes assessed; RR= relative risk; CI= confidence interval; IMT= intima-media thickness  Models adjusted for age, sex, and transport, occupational, and domestic physical activity | | | | |

TRANSPORT RELATED PHYSICAL ACTIVITY

| **Supplementary Table 14.** Associations between transport-related physical activity trajectories and continuous adult vascular structure and function outcomes. | | | | |
| --- | --- | --- | --- | --- |
| Outcome,  Latent transport-related physical activity trajectory | Model 1 | | Model 2 | |
|  |  | β (95% CI) |  | β (95% CI) |
| Carotid intima-media thickness (mm) | 914 |  | 853 |  |
| Persistently low |  | REF |  | REF |
| Increasingly high |  | 0.003 (-0.016, 0.023) |  | -0.001 (-0.021, 0.019) |
| Young’s Elastic Modulus (mmHg.mm) | 765 |  | 713 |  |
| Persistently low |  | REF |  | REF |
| High and Increasing |  | 5.96 (-21.38, 33.30) |  | 4.66 (-22.78, 32.11) |
| Carotid distensibility (%/10mmHg) | 765 |  | 713 |  |
| Persistently low |  | REF |  | REF |
| High and increasing |  | -0.01 (-0.11, 0.10) |  | -0.00 (-0.11, 0.11) |
| N = number of participants, 95%CI = 95% confidence interval  Model 1 = adjusted for age, sex, and leisure, occupational, and domestic physical activity  Model 2 = additionally adjusted for occupation-type, body mass index, smoking status, high-density lipoprotein, low-density lipoprotein, and index of relative socio-economic advantage and disadvantage. | | | | |

| **Supplementary Table 15.** Associations between transport-related physical activity trajectories among participants with complete case adult vascular structure and function outcomes | | | | |
| --- | --- | --- | --- | --- |
| Outcome,  Latent transport-related physical activity trajectory | Dichotomous | | Continuous | |
|  | n / N | RR (95% CI) | N | β (95% CI) |
| Young’s Elastic Modulus  (High-risk \| mmHg.mm) | 79 / 769 |  | 769 |  |
| Persistently low | 63 / 666 | REF | 666 | REF |
| High and increasing | 16 / 103 | 1.679 (0.862, 2.496) | 103 | 76.42 (-21.17, 34.01) |
| Carotid distensibility  (High-risk \| %/10mmHg) | 75 / 769 |  | 769 |  |
| Persistently low | 64 / 666 | REF | 666 | REF |
| High and increasing | 11 / 103 | 1.040 (0.429, 1.651) | 103 | -0.00 (-0.11, 0.10) |
| cIMT  (High-risk \| mm) | 91 / 769 |  | 769 |  |
| Persistently low | 77 / 666 | REF | 666 | REF |
| High and increasing | 14 / 103 | 1.175 (0.572, 1.779) | 103 | 0.01 (-0.02, 0.03) |
| Presence of carotid plaques | 107 / 769 |  |  |  |
| Persistently low | 92 / 666 | REF |  |  |
| High and increasing | 15 / 103 | 1.055 (0.529, 1.581) |  |  |
| n= number with high-risk outcomes; N= number with outcomes assessed; RR= relative risk; CI= confidence interval; IMT= intima-media thickness  Models adjusted for age, sex, and leisure, occupational, and domestic physical activity | | | | |

| **Supplementary Table 16.** Associations between transport-related physical activity trajectories and adult vascular structure and function outcomes among participants with complete case physical activity measures | | | | |
| --- | --- | --- | --- | --- |
| Outcome,  Latent transport-related physical activity trajectory | Dichotomous | | Continuous | |
|  | n / N | RR (95% CI) | N | β (95% CI) |
| Young’s Elastic Modulus  (High-risk \| mmHg.mm) | 37 / 382 |  | 382 |  |
| Persistently low | 32 / 339 | REF | 296 | REF |
| High and increasing | 5 / 43 | 1.252 (0.207, 2.297) | 86 | 7.40 (-34.66, 49.47) |
| Carotid distensibility  (High-risk \| %/10mmHg) | 35 / 382 |  | 382 |  |
| Persistently low | 29 / 339 | REF | 296 | REF |
| High and increasing | 6 / 43 | 1.519 (0.453, 2.585) | 86 | -0.05 (-0.21, 0.11) |
| Carotid IMT  (High-risk \| mm) | 47 / 477 |  | 477 |  |
| Persistently low | 43 / 425 | REF | 375 | REF |
| High and increasing | 4 / 52 | 0.750 (0.024, 1.476) | 102 | 0.01 (-0.01, 0.02) |
| Presence of carotid plaques | 47 / 432 |  |  |  |
| Persistently low | 43 / 382 | REF |  |  |
| High and increasing | 4 / 50 | 0.707 (0.014, 1.401) |  |  |
| n= number with high-risk outcomes; N= number with outcomes assessed; RR= relative risk; CI= confidence interval; IMT= intima-media thickness  Models adjusted for age, sex, and leisure, occupational, and domestic physical activity | | | | |

OCCUPATIONAL PHYSICAL ACTIVITY

| **Supplementary Table 17.** Associations between occupational physical activity trajectories and adult vascular structure and function outcomes among participants with complete case physical activity measures | | | | |
| --- | --- | --- | --- | --- |
| Outcome,  Latent occupational physical  activity trajectory | Dichotomous | | Continuous | |
|  | n / N | RR (95% CI) | N | β (95% CI) |
| Young’s Elastic Modulus  (High-risk \| mmHg.mm) | 37 / 382 |  | 382 |  |
| Persistently low | 32 / 329 | REF | 329 | REF |
| Low to high | 5 / 48 | 1.194 (0.174, 2.215) | 48 | 10.06 (-29.77, 49.89) |
| High and increasing | 0 / 5 | NA | 5 | -44, 01 (-152.00, 63.98) |
| Carotid distensibility  (High-risk \| %/10mmHg) | 35 / 382 |  | 382 |  |
| Persistently low | 28 / 329 | REF | 329 | REF |
| Low to high | 7 / 48 | 1.751 (0.663, 2.840) | 48 | -0.06 (-0.21, 0.08) |
| High and increasing | 0 / 5 | NA | 5 | 0.31 (-0.18, 0.80) |
| Carotid IMT  (High-risk \| mm) | 47 / 477 |  | 477 |  |
| Persistently low | 42 / 408 | REF | 408 | REF |
| Low to high | 5 / 62 | 0.790 (0.100, 1.479) | 62 | -0.01 (-0.04, 0.02) |
| High and increasing | 0 / 7 | NA | 7 | 0.05 (-0.03, 0.14) |
| Presence of carotid plaques | 47 / 432 |  |  |  |
| Persistently low | 41 / 373 | REF |  |  |
| Low to high | 6 / 54 | 1.008 (0.198, 1.818) |  |  |
| High and increasing | 0 / 5 | NA |  |  |
| n= number with high-risk outcomes; N= number with outcomes assessed; RR= relative risk; CI= confidence interval; IMT= intima-media thickness  Models adjusted for age, sex, and leisure, transport, and domestic physical activity | | | | |

DOMESTIC PHYSICAL ACTIVITY

| **Supplementary Table 18.** Associations between domestic physical activity trajectories and continuous adult vascular structure and function outcomes. | | | | |
| --- | --- | --- | --- | --- |
| Outcome,  Latent domestic physical activity trajectory | Model 1 | | Model 2 | |
|  |  | β (95% CI) |  | β (95% CI) |
| Carotid intima-media thickness (mm) | 914 |  | 853 |  |
| Persistently low |  | REF |  | REF |
| Consistently high |  | -0.006 (-0.023, 0.010) |  | -0.008 (-0.025, 0.008) |
| Young’s Elastic Modulus (mmHg.mm) | 765 |  | 713 |  |
| Persistently low |  | REF |  | REF |
| Consistently high |  | -3.22 (-25.67, 19.21) |  | -4.82 (-9.48, 16.91) |
| Carotid distensibility (%/10mmHg) | 765 |  | 713 |  |
| Persistently low |  | REF |  | REF |
| Consistently high |  | 0.01 (-0.08, 0.09) |  | 0.00 (-0.09, 0.09) |
| N = number of participants, 95%CI = 95% confidence interval  Model 1 = adjusted for age, sex, and leisure, transport, and occupational physical activity  Model 2 = additionally adjusted for occupation-type, body mass index, smoking status, high-density lipoprotein, low-density lipoprotein, and index of relative socio-economic advantage and disadvantage. | | | | |

| **Supplementary Table 19.** Associations between domestic physical activity trajectories and complete case adult vascular structure and function outcomes | | | | |
| --- | --- | --- | --- | --- |
| Outcome,  Latent domestic physical  activity trajectory | Dichotomous | | Continuous | |
|  | n / N | RR (95% CI) | N | β (95% CI) |
| Young’s Elastic Modulus  (High-risk \| mmHg.mm) | 79 / 769 |  | 769 |  |
| Persistently low | 62 / 600 | REF | 600 | REF |
| High and increasing | 17 / 169 | 1.679 (0.862, 2.496) | 169 | -2.95 (-25.59, 19.69) |
| Carotid distensibility  (High-risk \| %/10mmHg) | 75 / 769 |  | 769 |  |
| Persistently low | 58 / 600 | REF | 600 | REF |
| High and increasing | 17 / 169 | 1.040 (0.429, 1.652) | 169 | 0.00 (-0.08, 0.09) |
| Carotid IMT  (High-risk \| mm) | 91 / 769 |  | 769 |  |
| Persistently low | 79 / 600 | REF | 600 | REF |
| High and increasing | 12 / 169 | 0.694 (0.291, 1.098) | 169 | -0.00 (-0.02, 0.02) |
| Presence of carotid plaques | 107 / 769 |  |  |  |
| Persistently low | 79 / 600 | REF |  |  |
| High and increasing | 28 / 169 | 1.456 (0.678, 2.044) |  |  |
| n= number with high-risk outcomes; N= number with outcomes assessed; RR= relative risk; CI= confidence interval; IMT= intima-media thickness  Models adjusted for age, sex, and leisure, transport, and occupational physical activity | | | | |

| **Supplementary Table 20.** Associations between domestic physical activity trajectories and adult vascular structure and function outcomes among participants with complete case physical activity measures | | | | |
| --- | --- | --- | --- | --- |
| Outcome,  Latent domestic physical  activity trajectory | Dichotomous | | Continuous | |
|  | n / N | RR (95% CI) | N | β (95% CI) |
| Young’s Elastic Modulus  (High-risk \| mmHg.mm) | 37 / 382 |  | 382 |  |
| Persistently low | 30 / 296 | REF | 296 | REF |
| High and increasing | 7 / 86 | 1.252 (0.207, 2.297) | 86 | -2.71 (-34.20, 28.78) |
| Carotid distensibility  (High-risk \| %/10mmHg) | 35 / 382 |  | 382 |  |
| Persistently low | 27 / 296 | REF | 296 | REF |
| High and increasing | 8 / 86 | 1.519 (0.453, 2.585) | 86 | -0.00 (-0.12, 0.12) |
| Carotid IMT  (High-risk \| mm) | 47 / 477 |  | 477 |  |
| Persistently low | 40 / 375 | REF | 375 | REF |
| High and increasing | 7 / 102 | 0.779 (0.180, 1.379) | 102 | -0.02 (-0.04, 0.01) |
| Presence of carotid plaques | 47 / 432 |  |  |  |
| Persistently low | 37 / 336 | REF |  |  |
| High and increasing | 10 / 96 | 1.048 (0.352, 1.744) |  |  |
| n= number with high-risk outcomes; N= number with outcomes assessed; RR= relative risk; CI= confidence interval; IMT= intima-media thickness  Models adjusted for age, sex, and leisure, transport, and occupational physical activity | | | | |

**REFERENCES**

1. Raitakari OT, Juonala M, Kahonen M, Taittonen L, Laitinen T, Maki-Torkko N, et al. Cardiovascular risk factors in childhood and carotid artery intima-media thickness in adulthood: the Cardiovascular Risk in Young Finns Study. JAMA. 2003;290(17):2277-83.

2. Juonala M, Magnussen CG, Berenson GS, Venn A, Burns TL, Sabin MA, et al. Childhood Adiposity, Adult Adiposity, and Cardiovascular Risk Factors. N Engl J Med. 2011;365(20):1876-85.

3. Touboul PJ, Hennerici MG, Meairs S, Adams H, Amarenco P, Bornstein N, et al. Mannheim Carotid Intima-Media Thickness and Plaque Consensus (2004–2006–2011): An Update on Behalf of the Advisory Board of the 3rd, 4th and 5th Watching the Risk Symposia, at the 13th, 15th and 20th European Stroke Conferences, Mannheim, Germany, 2004, Brussels, Belgium, 2006, and Hamburg, Germany, 2011. Cerebrovascular Diseases. 2012;34(4):290-6.

4. Huynh QL, Blizzard CL, Raitakari O, Sharman JE, Magnussen CG, Dwyer T, et al. Vigorous physical activity and carotid distensibility in young and mid-aged adults. Hypertension Research. 2015;38(5):355-60.

5. Buscot MJ, Thomson RJ, Juonala M, Sabin MA, Burgner DP, Lehtimaki T, et al. Distinct child-to-adult body mass index trajectories are associated with different levels of adult cardiometabolic risk. European Heart Journal. 2018;39(24):2263-70.

6. Evans JT, Buscot M-J, Gall S, Dwyer T, Venn A, Cleland V. Distinct patterns of adult transport-related physical activity (TRPA) behaviour exist independent of the TRPA behaviours of childhood: the childhood determinants of adult health study. International Journal of Behavioral Nutrition and Physical Activity. 2023;20(1):63.

7. Evans JT, Phan H, Buscot MJ, Gall S, Cleland V. Correlates and determinants of transport-related physical activity among adults: An interdisciplinary systematic review. BMC Public Health. 2022;22(1):1-26.

8. Bauman AE, Reis RS, Sallis JF, Wells JC, Loos RJ, Martin BW, et al. Correlates of physical activity: why are some people physically active and others not? The Lancet. 2012;380(9838):258-71.

9. Wardenaar K. Latent Class Growth Analysis and Growth Mixture Modeling using R: A tutorial for two R-packages and a comparison with Mplus2020.

10. Proust-Lima C, Philipps V, Liquet B. Estimation of extended mixed models using latent classes and latent processes: The R package lcmm. Journal of Statistical Software. 2017;78(2):1 - 56.
